# Intestinal angina in a patient with hypertrophic obstructive cardiomyopathy: a case report

**DOI:** 10.1186/s13256-016-1055-8

**Published:** 2016-09-29

**Authors:** Takuto Hamaoka, Wataru Omi, Yoshiteru Sekiguti, Shigeo Takata, Shuichi Kaneko, Oto Inoue, Shinichiro Takashima, Hisayoshi Murai, Soichiro Usui, Takeshi Kato, Hiroshi Furusho, Masayuki Takamura

**Affiliations:** 1 Department of Disease Control and Homeostasis, Graduate School of Medical Science, Kanazawa University, 13-1 Takara-machi, Kanazawa, 920-8641 Japan; 2 Department of Cardiology, Kanazawa Municipal Hospital, Kanazawa, Japan

**Keywords:** Intestinal angina, Hypertrophic obstructive cardiomyopathy, Advanced atrioventricular block, Case report

## Abstract

**Background:**

Intestinal angina is characterized by recurrent postprandial abdominal pain and anorexia. Commonly, these symptoms are caused by severe stenosis of at least two vessels among the celiac and mesenteric arteries. However, intestinal perfusion is affected not only by the degree of arterial stenosis but also by systemic perfusion. We experienced a unique case of intestinal angina caused by relatively mild stenosis of the abdominal arteries complicated with hypertrophic obstructive cardiomyopathy.

**Case presentation:**

We report an 86-year old Japanese man with hypertrophic obstructive cardiomyopathy and advanced atrioventricular block who was diagnosed with intestinal angina. Computed tomography showed mild stenosis of the celiac artery and severe stenosis of the inferior mesenteric artery, and these lesions were relatively mild compared with other reports. A dual-chamber pacemaker with right ventricular apical pacing was implanted to improve the obstruction of the left ventricular outflow tract. After implantation, the patient’s abdominal symptoms diminished markedly, and improvement of the left ventricular outflow tract obstruction was observed.

**Conclusions:**

Although intestinal angina is generally defined by severe stenosis of at least two vessels among the celiac and mesenteric arteries, the present case suggests that hemodynamic changes can greatly affect intestinal perfusion and induce intestinal angina in the presence of mild stenosis of the celiac and mesenteric arteries.

## Background

Intestinal angina is caused by chronic intestinal ischemia characterized by recurrent postprandial abdominal pain and a decrease in body weight with anorexia [1]. The symptom is commonly manifested when at least two vessels of the celiac and mesenteric arteries have developed moderate to severe stenosis or occlusion. Hypertrophic obstructive cardiomyopathy (HOCM) is associated with a reduction in cardiac output and systemic perfusion fluctuated by multiple hemodynamic conditions [2] such as hypovolemia or high blood pressure. Hypovolemic conditions lead to a reduction in left ventricular volume and the narrowing or collapse of the left ventricular outflow tract (LVOT), resulting in an increased LVOT pressure gradient. On the other hand, hypertension increases the cardiac afterload, which inhibits the collapse of LVOT and results in a decreased LVOT pressure gradient [3].

In this report, we describe a unique case of intestinal angina complicated with HOCM and abruptly worsened by advanced atrioventricular (AV) block in the presence of mild stenosis of the abdominal arteries.

## Case presentation

An 86-year old Japanese man was admitted to our hospital because of worsening postprandial abdominal pain, anorexia, and general malaise. He was previously diagnosed with HOCM on cardiac magnetic resonance imaging and echocardiography, but because he was asymptomatic, he was not taking oral medications. He had been suffering from repetitive and paroxysmal upper abdominal pain for 1 year. His symptoms occurred approximately 15 minutes after eating and usually disappeared within 1 hour and were sometimes accompanied by watery diarrhea. Once his abdominal attacks occurred, he had great difficulties with oral intake due to postprandial pain, but his symptoms disappeared upon fluid replacement for several days. He presented to our department because of worsening and more sustained symptoms within the week prior.

**Fig. 1:**
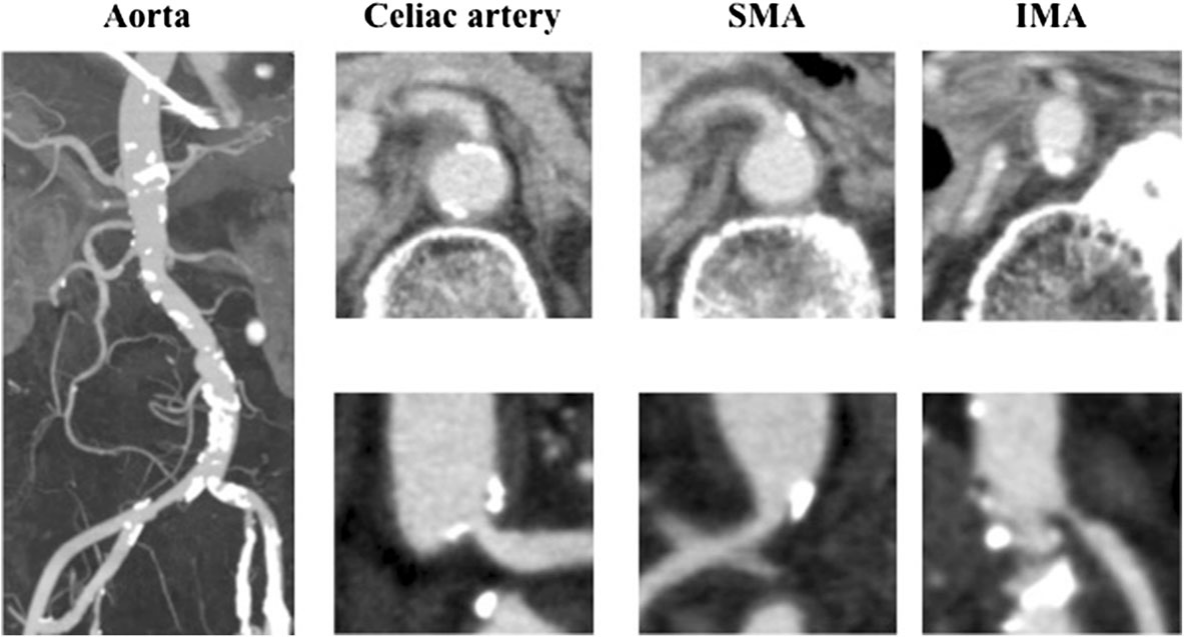
Enhanced computed tomography showed severe atherosclerosis of the arteries. The aorta showed broad calcifications, and the celiac artery showed moderate stenosis, although the lumen of the SMA was relatively patent. In addition, stenosis of the IMA was very severe. *IMA* inferior mesenteric artery, *SMA* superior mesenteric artery

**Fig. 2:**
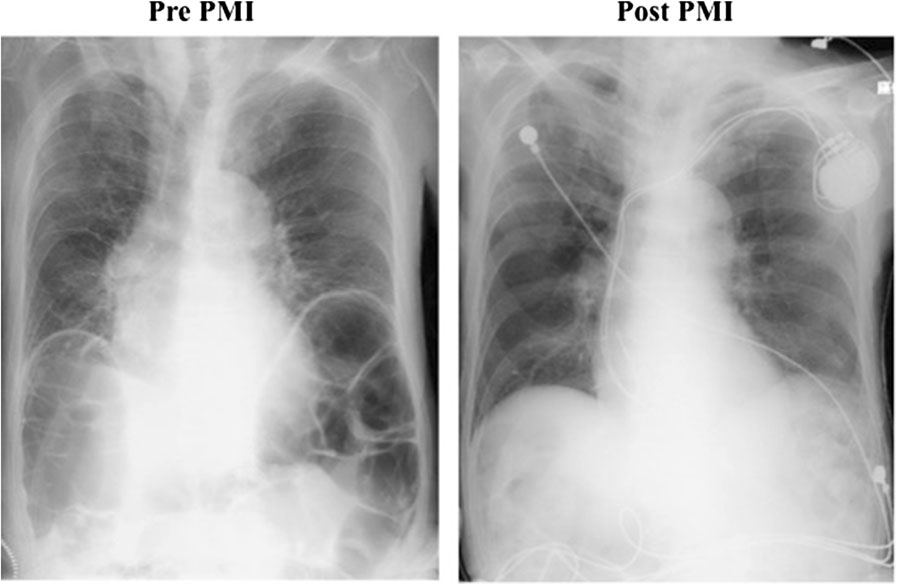
Chest X-ray examinations pre-PMI demonstrated significant dilatation of the bowels; however, this dilatation was improved after PMI with right ventricular apical pacing. *PMI* pacemaker implantation

He was emaciated and exhausted and had lost 7 kg in the last 1 year. A physical examination showed a low blood pressure (90/60 mmHg) and bradycardia (40 beats per minute). He had no signs of disorientation or paralysis. A holosystolic ejection murmur at the fourth left sternal border was auscultated, and abdominal palpitation showed no abnormal findings. The murmur was accentuated by the Valsalva maneuver and walking. Laboratory data showed slight anemia (hemoglobin 11.5 g/day), hypoalbuminemia (3.8 g/dL, normal range 3.8–5.3 g/dL), and mild renal dysfunction (serum creatinine (Cre) 1.33 mg/dL, normal range 0.6–1.2 mg/dL; blood urea nitrogen (BUN) 39 mg/dL, normal range 9–23 mg/dL; estimated glomerular filtration rate (eGFR) 39.5 mL/minute/1.73 m^2^), and no signs of dyslipidemia or diabetes mellitus (low density lipoprotein cholesterol 98 mg/dL; glycosylated hemoglobin 5.3 %; fasting blood glucose 100 mg/dL). Our patient’s activated partial thromboplastin time (33.1 seconds) and prothrombin time (13.0 seconds) were within the normal ranges. Gastroscopy and colonoscopy examinations detected no abnormal findings, while a computed tomography (CT) scan with contrast enhancement demonstrated moderate stenosis of the celiac artery and severe stenosis of the inferior mesenteric artery (Fig. 1). In addition, significant dilatation of the bowels was observed by CT and chest X-ray examinations (Fig. 2).

**Fig. 3:**
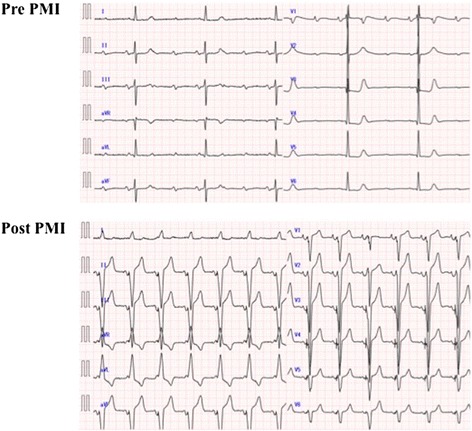
An electrocardiogram pre-PMI showed severe bradycardia with advanced AV block (approximately 40 beats/minute). After PMI, bradycardia was improved. *AV* atrioventricular, *PMI* pacemaker implantation

**Fig. 4:**
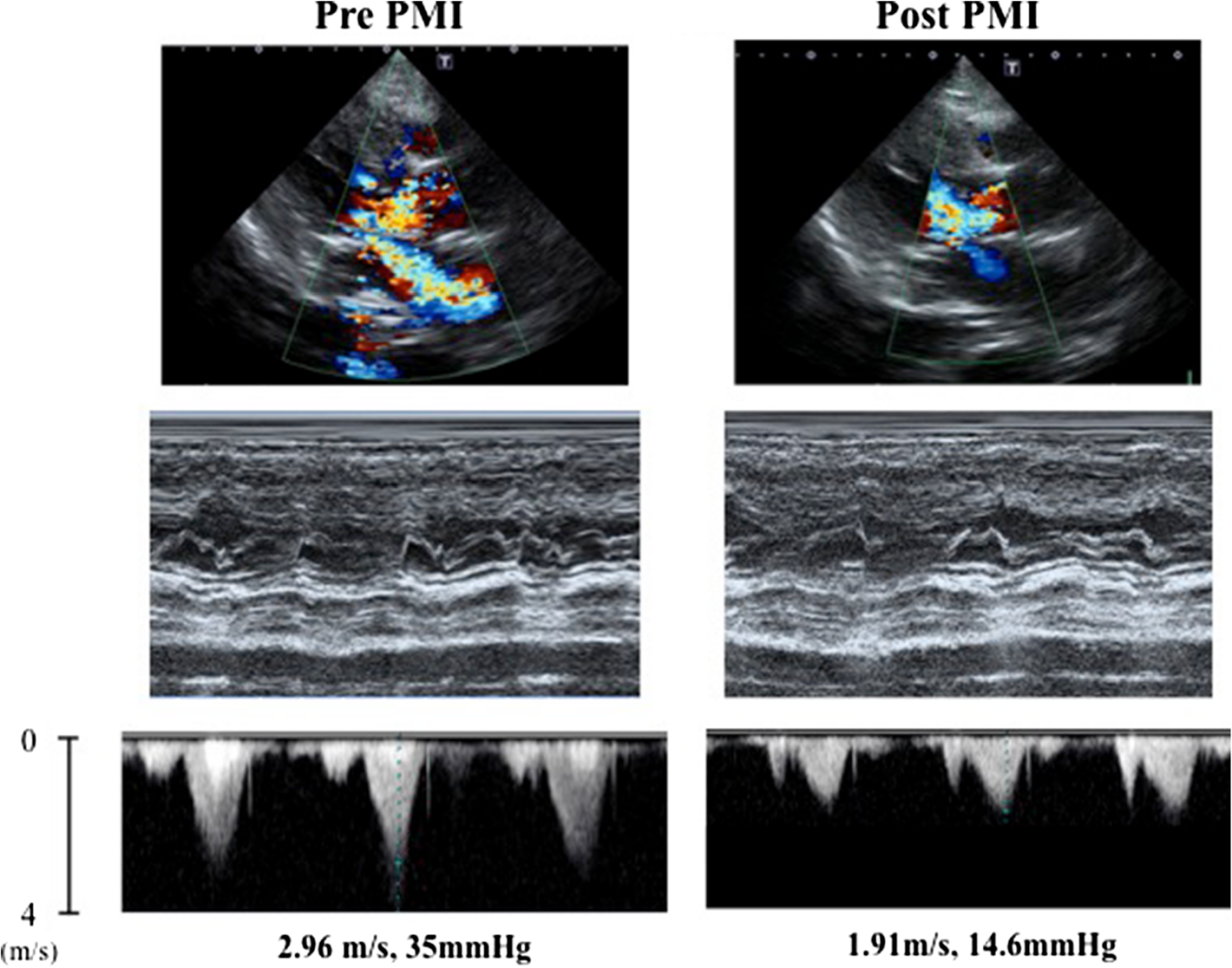
Transthoracic echocardiography demonstrated severe MR with SAM and LVOT obstruction (LVOT pressure gradient, 35 mmHg) pre-PMI. After PMI, transthoracic echocardiography showed significant improvements in MR and LVOT obstruction (LVOT pressure gradient, 14.6 mmHg). *LVOT* left ventricular outflow tract, *MR* mitral valve regurgitation, *SAM* systolic anterior motion

An electrocardiogram revealed advanced AV block and left high voltage with ST-T change; AV disturbance had not been detected previously (Fig. 3). Transthoracic echocardiography showed significant obstruction of the LVOT (pressure gradient, 35 mmHg) and mitral regurgitation with systolic anterior motion of the anterior mitral leaflet (Fig. 4). These echocardiographic findings were similar to those of echocardiography performed the previous year on our patient.

CT angiography was classified as mild to account for our patient’s severe postprandial pain, since our patient’s superior mesenteric artery, which had the widest intestinal perfusion area, was patent in this patient; however, in many previous reports concerning intestinal angina, stenosis of this artery was demonstrated, and angioplasty was performed [4]. Thus, it was suspected that hemodynamic failure contributed to the pathophysiology. That is, under the chronic low output condition of uncontrolled HOCM, factors such as occasional dehydration and bradycardia had reduced systemic perfusion, especially that of intestinal blood flow. We performed a pacemaker implantation with right ventricular apical pacing to improve our patient’s hemodynamic condition. After dual-chamber pacemaker implantation, pacing AV delay was optimized for a minimum LVOT pressure gradient and mitral valve regurgitation under echocardiographic estimations (AV delay 100 milliseconds for LVOT 14.6 mmHg, Fig. 4). Following these procedures, our patient’s appetite and daily activity improved dramatically. In addition, his blood pressure improved significantly, his renal function became normal (Cre, 0.83 mg/dL; BUN, 25 mg/dL; eGFR, 66.2 mL/minute/1.73 m^2^), and a chest X-ray examination showed significant improvement of intestinal dilatation (Fig. 2), suggesting increase in systemic and intestinal perfusion. Finally, he has been completely free from abdominal symptoms in the last 3 months.

## Discussion

Classically, chronic intestinal angina is caused by a reduction in mesenteric blood flow [1], and the pathophysiology of most cases is atherosclerotic stenosis of the celiac and mesenteric arteries. Arterial dissection, fibromuscular dysplasia, and vasculitis are included as rare etiologies of arterial narrowing, and the median arcuate ligament of the diaphragm can compress the celiac artery and disturb blood flow (median arcuate ligament syndrome) [5, 6]. Intestinal circulation consists of an abundant collateral blood supply, and chronic intestinal ischemia is associated with high-grade stenosis or occlusion of two or more of the three major vessels: the celiac artery, the superior and inferior mesenteric arteries [7, 8]. In our case, the arterial lesions were relatively mild compared with previous reports [9, 10]; thus, we hypothesized that our patient’s major symptoms were not due to his arterial lesions alone.

Specifically, our patient’s symptoms depended greatly on his hemodynamic condition, which caused a flow discrepancy between demand and supply [1]. Once an abdominal attack occurred, our patient’s blunted oral intake exacerbated repetitive postprandial attacks; only fluid replacement led to remission. Furthermore, advanced AV block abruptly worsened his condition and pacemaker implantation with right ventricular apical pacing resulted in complete remission of his symptoms. In patients with HOCM, dehydration augments LVOT obstruction and mitral regurgitation, leading to reduced cardiac output [2]. Pacemaker implantation with right ventricular apical pacing increases cardiac output by optimizing chronotropic action as well as by lowering the LVOT gradient in HOCM patients [11, 12]. Nonocclusive mesenteric ischemia is a type of intestinal ischemia attributed to hemodynamic failure, sometimes without arterial stenosis or occlusion. Vasospasm of mesenteric vessels is assumed to be a major pathophysiological and acute homeostatic response used to maintain systemic circulation at the expense of the splanchnic blood supply in critically ill conditions, such as cardiogenic shock [13]. In our case, the patient complained mainly of long-standing postprandial abdominal pain without any signs of severe circulatory failure in other organs. He was later diagnosed with uncontrolled HOCM complicated with stenosis of the celiac and mesenteric arteries, and his symptoms reflected excessive hemodynamic fluctuation specific to HOCM. Generally, easily digestible meals or vasodilators are used as supportive therapy, while the definitive treatment for intestinal angina is revascularization by surgery or catheterization [13]. However, our patient was too old and emaciated to undergo these invasive treatments. Although he underwent pacemaker implantation, it was less invasive than was revascularization therapy. HOCM is commonly treated with oral medications, such as calcium channel blockers, β-adrenoreceptor blockers, and antiarrhythmic agents included in group Ia of the Vaughan-Williams criteria, to reduce myocardial oxygen consumption and inhibit left ventricular hypercontraction. Percutaneous transluminal septal myocardial ablation, septal myectomy, and pacemaker implantation are other potential treatments if oral therapy is not successful. Although the efficacy of septal myectomy in improving the long-term prognosis of HOCM patients was reported previously [14], this treatment is well known for its high mortality rate, with older age being a risk factor [15]. In this case, pacemaker implantation was selected as the treatment, because our patient’s systemic circulation and abdominal symptoms were clearly exacerbated by bradycardia from AV block.

Some limitations were observed in this case report. No intestinal hemodynamic parameters were evaluated, because abdominal ultrasound was unable to detect the celiac and mesenteric arterial flows. Furthermore, our patient was very emaciated and unable to undergo catheter examination. However, after pacemaker implantation, his symptoms, intestinal dilatation, and renal function improved without fluid replacement or additional medications, which reflected improvement of his systemic circulation.

## Conclusions

This case suggests that hemodynamic changes could greatly affect intestinal perfusion in the presence of mild stenosis of the celiac and mesenteric arteries. Thus, it should be recognized that intestinal angina might be a symptom of cardiovascular disease, especially cardiac failure.

## Abbreviations

AV: atrioventricular
BUN: blood urea nitrogen
Cre: creatinine
CT: computed tomography
eGFR: estimated glomerular filtration rate
HOCM: hypertrophic obstructive cardiomyopathy
LVOT: left ventricular outflow tract
